# The Atlanta Polyclinic

**Published:** 1893-02

**Authors:** 


### THE ATLANTA POLYCLINIC.

We take pleasure in directing the attention of our readers to the advertisement of the Atlanta Polyclinic which appears in this issue. The organization of this school has long been in contemplation, from the recognized need in this section of an institution for post-graduate work, with special attention to clinical instruction. The faculty is composed of prominent Atlanta physicians who are thoroughly interested in the work and who will do all in their power to make it a success. The new enterprise has our sincere good wishes.

THE Sanitarium of Dr. J. B. S. Holmes, at Rome, was totally destroyed by fire on the morning of January 27th. Loss, $100,000; insurance about $65,000. Many patients were in the building when the fire was discovered, but all were removed. Dr. Holmes had built up an excellent and flourishing institution at Rome, and we much regret his misfortune. He will continue his work, and has already arranged for suitable buildings in which to accommodate his patients.

